# Supplementary material for: Flanking sequences influence the activity of TET1 and TET2 methylcytosine dioxygenases and affect genomic 5hmC patterns
Source: Commun Biol. 2022 Jan 24;5:92. doi: 10.1038/s42003-022-03033-4 (PMC8786823; doi:10.1038/s42003-022-03033-4)
Supplement: Supplementary file 2 — Supplementary information [file 42003_2022_3033_MOESM2_ESM.pdf]

Flanking sequences influence the activity of TET1 and TET2 methylcytosine dioxygenases and affect genomic 5hmC patterns

Sabrina Adam, Julia Bräcker, Viviane Klingel, Bernd Osteresch, Nicole E. Radde, Jens Brockmeyer, Pavel Bashtrykov, Albert Jeltsch

Supplementary information

Supplementary Tables

Supplementary Table 1: Summary of the NGS sequencing data used in this study.

Supplementary Table 2: Results of no-enzyme control reactions.

Supplementary Table 3: Oligonucleotides and primers used for the Deep Enzymology experiments.

Supplementary Table 4: Oligonucleotides used for the TET oxidation kinetics and UHRF2 SRA DNA binding studies.

Supplementary Figures

Supplementary Figure 1: Terminology and principles of the Deep Enzymology approach.

Supplementary Figure 2: Compilation of the correlation of mCG oxidation flank preferences.

Supplementary Figure 3: Compilation of the correlation of hmCG oxidation flank preferences.

Supplementary Figure 4: Determination of reaction rates for oxidation of 5mC and 5hmC in NNCGNN context.

Supplementary Figure 5: Correlation of the flanking sequence preferences of TET1, TET2, DNMT1, DNMT3A and DNMT3B.

Supplementary Figure 6: Additional data regarding the LC-MS TET activity assays.

Supplementary Figure 7: Fitting of kinetic data requires a processive reaction step.

Supplementary Figure 8: Compilation of the correlation of mCpX oxidation flank preferences.

Supplementary Figure 9: Comparison of genomic NNCGNN 5hmC patterns in lung and liver cells revealing very high similarity.

Supplementary Figure 10: Schematic drawing of the TET1 and TET2 enzyme constructs used in this study.

Supplementary Figure 11: Uncropped images of Figure 3d and e.

Supplementary References

Supplementary Tables

Supplementary Table 1: Summary of the NGS sequencing data used in this study.

Substrate	Enzyme	Repeat	c(TET) [µM]	total reads	sum
5mCG	TET1	R1	1.3	28868	68539
		R2	1.3	14109	
		R3	1.3	25562	
	TET2 V1	R1	2	41124	178607
		R2	3	14766	
		R3	4	15337	
		R4	3	50336	
		R5	4	57044	
	TET2 V2	R1	1.5	68389	178980
		R2	2.3	66053	
		R3	3	44538	
	no enzyme	R1	0	144545	144545
5hmCG	TET1	R1	2.6	92060	461761
		R2	2.6	99216	
		R3	2.3	82171	
		R4	2	93378	
		R5	2	94936	
	TET2 V1	R1	4.4	85375	133495
		R2	4.4	48120	
	TET2 V2	R1	4	43315	111154
		R2	4	67839	
	no enzyme	R1	0	182237	182237
5mCX	TET1	R1	1.3	65996	140267
		R2	1.3	74271	
	TET2 V2	R1	3	107425	207921
		R2	3	100496	
	no enzyme	R1	0	34354	34354

Supplementary Table 2: Results of no-enzyme control reactions. No-enzyme control reactions were conducted with the hemimethylated and hemihydroxymethylated CpG substrates. In each case, readout of C was expected in the upper DNA strand after bisulfite conversion and readout of T in the lower DNA strand. The data confirm low rates of "overconversion" leading to T readout in the upper strand (0.3 %) and low rates of incomplete conversion leading to C readout in the lower strand (1-2 %).

Substrate	Strand	Original base	Readout	
			C (%)	T (%)
5mCG	upper	5mC	98.986	1.014
	lower	C	0.338	99.662
5hmCG	upper	5hmC	97.967	2.033
	lower	C	0.302	99.698
5mCX	upper	5mC	98.274	1.726
	lower	C	0.342	99.758

Supplementary Table 3: Oligonucleotides and primers used for the Deep Enzymology experiments.

Oligo	Sequence
HM rand.	GAGTGTGACTAGGCTCTCACTGCCNNNNNNNNNN mC GNNNNNNNNNNGAGAGGAGACCTAGTGAGAAG
OH rand.	GAGTGTGACTAGGCTCTCACTGCCNNNNNNNNNN hmC GNNNNNNNNNNGAGAGGAGACCTAGTGAGAAG
CH rand.	GAGTGTGACTAGGCTCTCACTGCCNNNNNNNNNN mC HNNNNNNNNNNGAGAGGAGACCTAGTGAGAAG
Extension primer	CTTCTCACTAGGTCTCC
Hairpin	pGAGAAGGGATGTGGATACACATCCCT
PCR1 fwd	ACACTCTTTCCCTACACGACGCTCTTCCGATCTNNNNNNATAGAGTGTGATTAGGTTTTTATTGTT
PCR1 rev	GTGACTGGAGTTCAGACGTGTGCTCTTCCGATCTNNNNNNTACAAATATAACTAAACTCTCACTAAC
PCR2 fwd	AATGATACGGCGACCACCGAGATCTACACATTACTCGACACTCTTTCCCTACACGACGCTCTTCCGATCT
PCR2 rev	AATGATACGGCGACCACCGAGATCTACACTCCGGAGAACACTCTTTCCCTACACGACGCTCTTCCGATCT

Exemplary barcode and index parts are underlined. N refers to random nucleotides.

Supplementary Table 4: Oligonucleotides used for the TET oxidation kinetics and UHRF2 SRA DNA binding studies.

**Oligo**	**Sequence**
TET-pref	GAAGCTGGGACTTACGTAAGGAGAGTGCAA
TET-pref-mC	GAAGCTGGGACTTA mC GTAAGGAGAGTGCAA
TET-pref-hmc	GAAGCTGGGACTTA hmC GTAAGGAGAGTGCAA
TET-pref-rev	TTGCACTCTCCTTACGTAAGTCCCAGCTTC
TET-disf	GAAGCTGGGACGCGCGCCGGGAGAGTGCAA
TET-disf-mC	GAAGCTGGGACGCG mC GCCGGGAGAGTGCAA
TET-disf-hmc	GAAGCTGGGACGCG hmC GCCGGGAGAGTGCAA
TET-disf-rev	TTGCACTCTCCCGGCGCGCGTCCCAGCTTC

Supplementary Figures

Supplementary Figure 1: Terminology and principles of the Deep Enzymology approach. a Terminology used in this document to describe the DNA substrate. The target sites of the TET enzymes include CpG and non-CpG sites. Target residues for oxidation are either 5mC or 5hmC. **b** Principle workflow of the Deep Enzymology approach [1]. Second strand synthesis using synthetic oligonucleotides with mCpG site in random N_{10} flank context as template (step 1). Oxidation of the pool of sequences by TET enzymes (step 2). Hairpin ligation (step 3) and bisulfite conversion (step 4). Generation of sequencing libraries by addition of barcodes and indices (Step 5), and Illumina sequencing (step 6). Extraction of original sequences and cytosine oxidation state followed by downstream analysis (step 7). **c** Principle of the detection of 5-methylcytosine oxidation by bisulfite conversion. The scheme indicates the different oxidation states of 5-methylcytosine and its readout after bisulfite conversion illustrating that the transition of 5hmC to 5fC is detected by this technology.

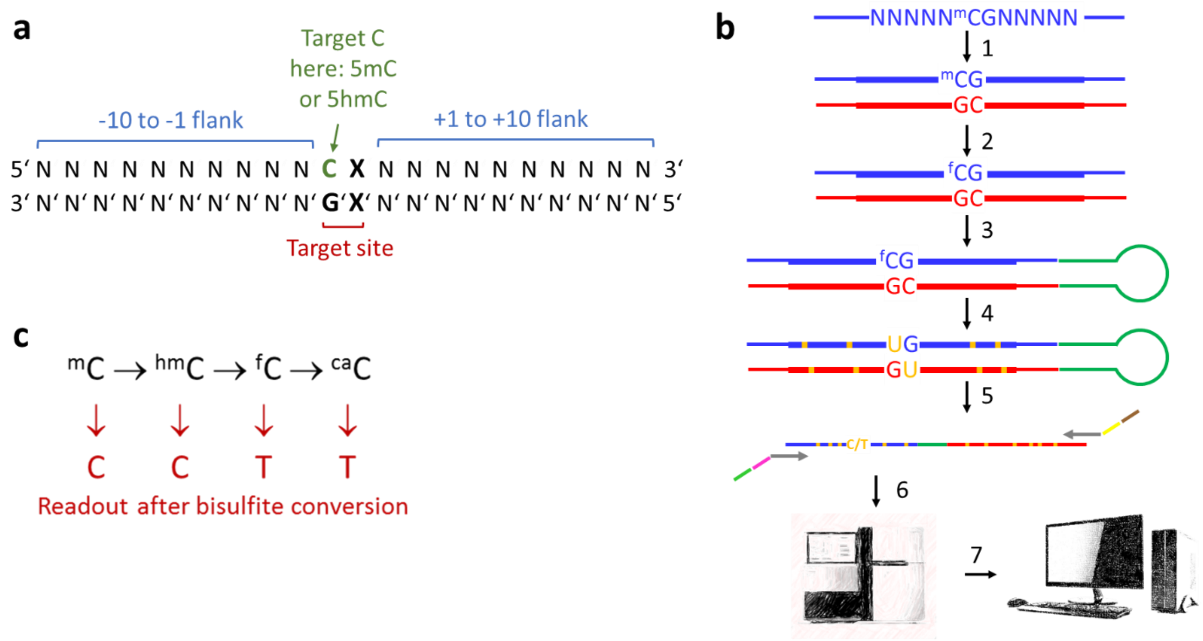

Supplementary Figure 2: Compilation of the correlation of mCG oxidation flank preferences. a Correlation of NNmCGNN flank preferences observed in the individual experimental repeats with TET1, TET2 V1 and TET2 V2. **b** Heatmap of the averaged oxidation activities of TET1, TET2 V1 and TET2 V2 at NNmCGNN sites sorted by the average activity. The heatmap scale is between zero and the relative maximum of each sample. **c** Correlation of the averaged NNmCGNN flank preferences of TET1, TET2 V1 and TET2 V2. In panels a and c, Pearson correlation factors are shown. Note, the very high similarity of TET2 V1 and TET2 V2.

a

		TET1			TET2 V1					TET2 V2		
		R1	R2	R3	R1	R2	R3	R4	R5	R1	R2	R3
TET1	R1	1.00	0.86	0.94	0.82	0.73	0.79	0.77	0.77	0.81	0.87	0.87
	R2	0.86	1.00	0.87	0.77	0.73	0.75	0.74	0.75	0.77	0.79	0.76
	R3	0.94	0.87	1.00	0.83	0.77	0.80	0.81	0.79	0.84	0.87	0.85
TET2 V1	R1	0.82	0.77	0.83	1.00	0.92	0.94	0.96	0.96	0.97	0.95	0.91
	R2	0.73	0.73	0.77	0.92	1.00	0.91	0.93	0.94	0.94	0.87	0.81
	R3	0.79	0.75	0.80	0.94	0.91	1.00	0.94	0.95	0.95	0.92	0.87
	R4	0.77	0.74	0.81	0.96	0.93	0.94	1.00	0.97	0.98	0.92	0.86
	R5	0.77	0.75	0.79	0.96	0.94	0.95	0.97	1.00	0.97	0.91	0.86
TET2 V2	R1	0.81	0.77	0.84	0.97	0.94	0.95	0.98	0.97	1.00	0.95	0.90
	R2	0.87	0.79	0.87	0.95	0.87	0.92	0.92	0.91	0.95	1.00	0.98
	R3	0.87	0.76	0.85	0.91	0.81	0.87	0.86	0.86	0.90	0.98	1.00

b

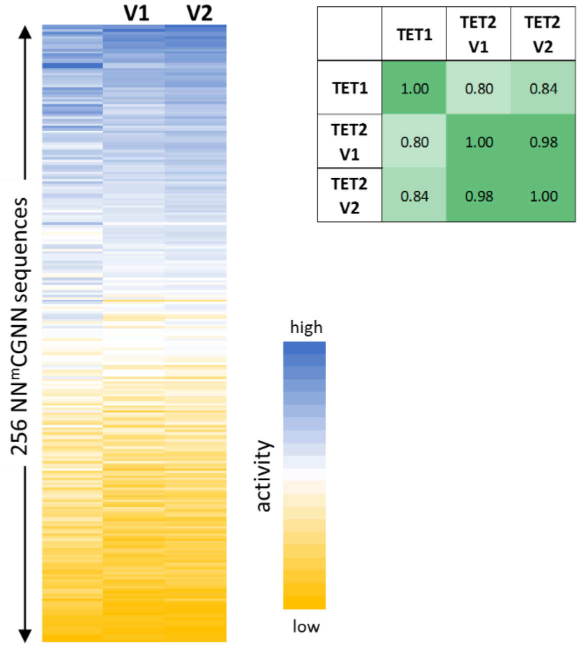

c

	TET1	TET2 V1	TET2 V2
TET1	1.00	0.80	0.84
TET2 V1	0.80	1.00	0.98
TET2 V2	0.84	0.98	1.00

Supplementary Figure 3: Compilation of the correlation of hmCG oxidation flank preferences. a Correlation of NNhmCGNN flank preferences observed in the individual experimental repeats with TET1, TET2 V1 and TET2 V2. **b** Heatmap of the averaged oxidation activities of TET1, TET2 V1 and TET2 V2 at NNhmCGNN sites sorted by the average activity. The heatmap scale is between zero and the relative maximum of each sample. **c** Correlation of the averaged NNmCGNN (taken from Supplementary Figure 2) and NNhmCGNN flank preferences of TET1, TET2 V1 and TET2 V2. In a and c Pearson correlation factors are shown. Note, the very high similarity of TET2 V1 and TET2 V2.

a

		TET1					TET2 V1		TET2 V2	
		R1	R2	R3	R4	R5	R1	R2	R1	R2
TET1	R1	1.00	0.97	0.97	0.96	0.96	0.86	0.82	0.85	0.85
	R2	0.97	1.00	0.97	0.96	0.97	0.85	0.83	0.85	0.86
	R3	0.97	0.97	1.00	0.98	0.98	0.87	0.84	0.87	0.86
	R4	0.96	0.96	0.98	1.00	0.98	0.87	0.84	0.86	0.86
	R5	0.96	0.97	0.98	0.98	1.00	0.86	0.84	0.86	0.85
TET2 V1	R1	0.86	0.85	0.87	0.87	0.86	1.00	0.97	0.98	0.96
	R2	0.82	0.83	0.84	0.84	0.84	0.97	1.00	0.97	0.94
TET2 V2	R1	0.85	0.85	0.87	0.86	0.86	0.98	0.96	1.00	0.97
	R2	0.85	0.86	0.86	0.86	0.85	0.97	0.94	0.97	1.00

b

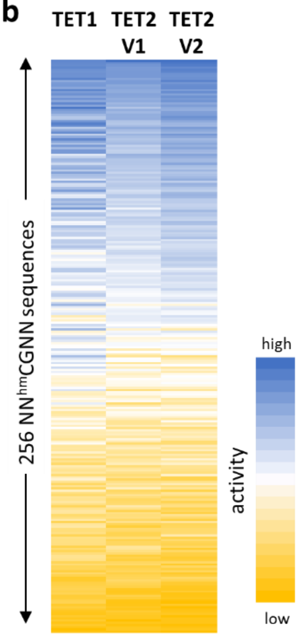

c

		TET1		TET2 V1		TET2 V2	
		5mC	5hmC	5mC	5hmC	5mC	5hmC
TET1	5mC	1.00	0.94	0.80	0.78	0.84	0.79
	5hmC	0.94	1.00	0.83	0.87	0.87	0.88
TET2 V1	5mC	0.80	0.83	1.00	0.94	0.98	0.93
	5hmC	0.78	0.87	0.94	1.00	0.96	0.98
TET2 V2	5mC	0.84	0.87	0.98	0.96	1.00	0.96
	5hmC	0.79	0.88	0.93	0.98	0.96	1.00

Supplementary Figure 4: Determination of reaction rates for oxidation of 5mC and 5hmC in NNCGNN context. The figures show examples of the primary data and fit curves.

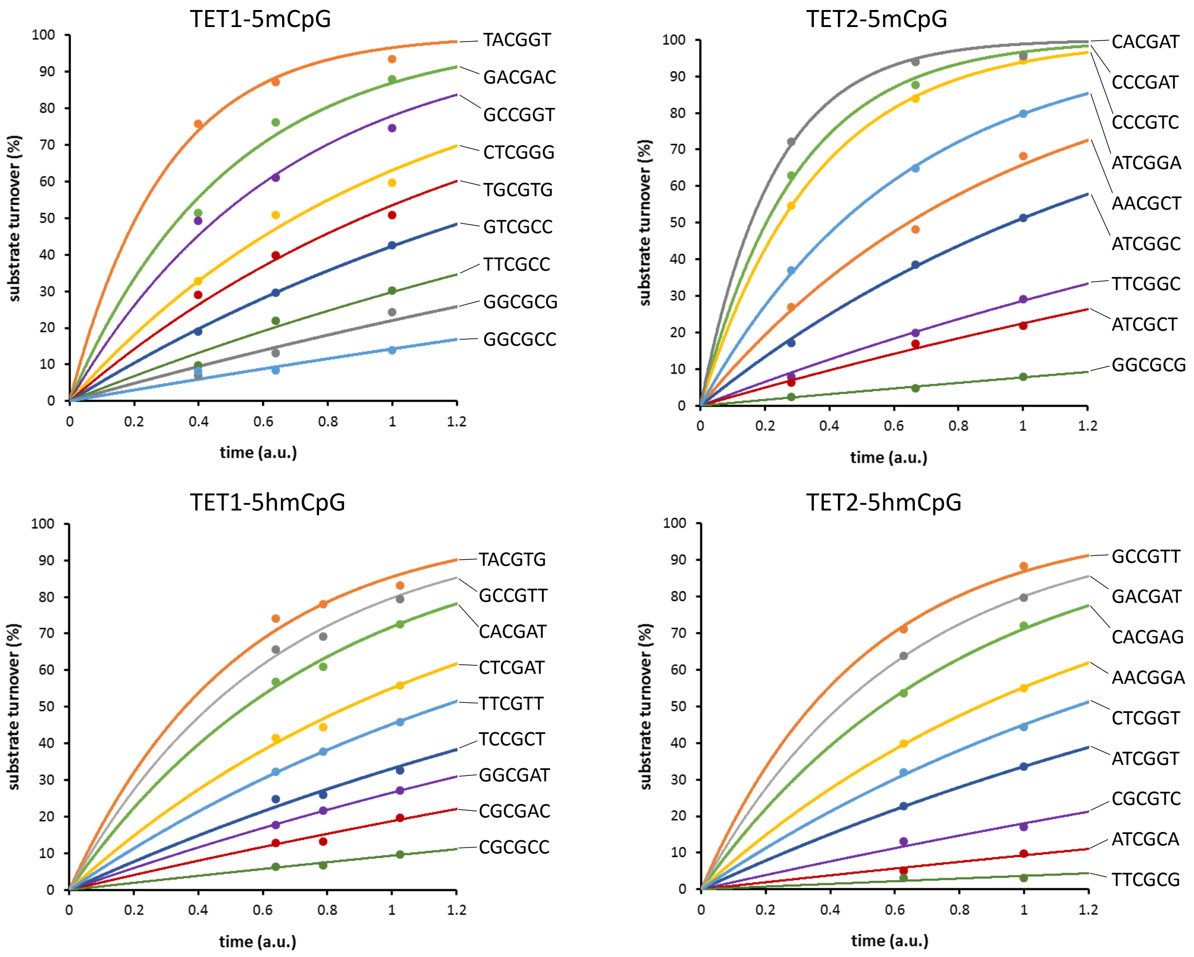

Supplementary Figure 5: Correlation of the flanking sequence preferences of TET1, TET2, DNMT1, DNMT3A and DNMT3B. Pearson correlation factors of the TET1 and TET2 5mCG oxidation NNCGNN flanking sequence preferences determined here with previously published CpG methylation preferences of DNMT1 [2] and DNMT3A and DNMT3B [3].

	TET1	TET2	DNMT1	DNMT3A	DNMT3B
**TET1**	1.00	0.84	0.26	0.10	0.32
**TET2**	0.84	1.00	0.17	0.04	0.33
**DNMT1**	0.26	0.17	1.00	0.06	-0.05
**DNMT3A**	0.10	0.04	0.06	1.00	0.36
**DNMT3B**	0.32	0.33	-0.05	0.36	1.00

Supplementary Figure 6: Additional data regarding the LC-MS TET activity assays. a Characteristic ion transitions used in this work. **b** Examples of calibration curves determined for 5mdC, 5hmdC, 5fdC and 5cadC. **c** Examples of LC profiles of standard peaks and peaks obtained from TET oxidation reactions.

a

Nucleoside	Precursor ion [m/z]	Product ion [m/z]
dA	252.1097	136.0623
dC	228.0984	112.0511
dG	268.1046	152.0572
dT	243.0981	127.0508
5mdC	242.1140	126.0660
5hmdC	258.1090	142.0617
5fdC	256.0930	140.0460
5cadC	272.0880	156.0410

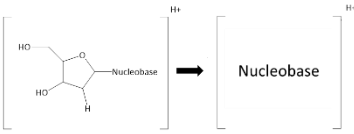

b

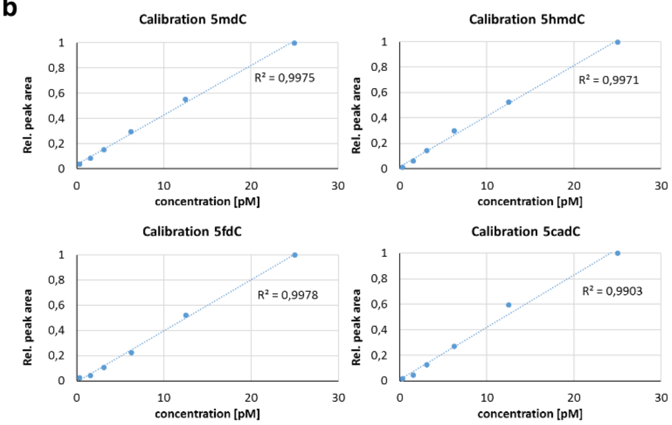

c

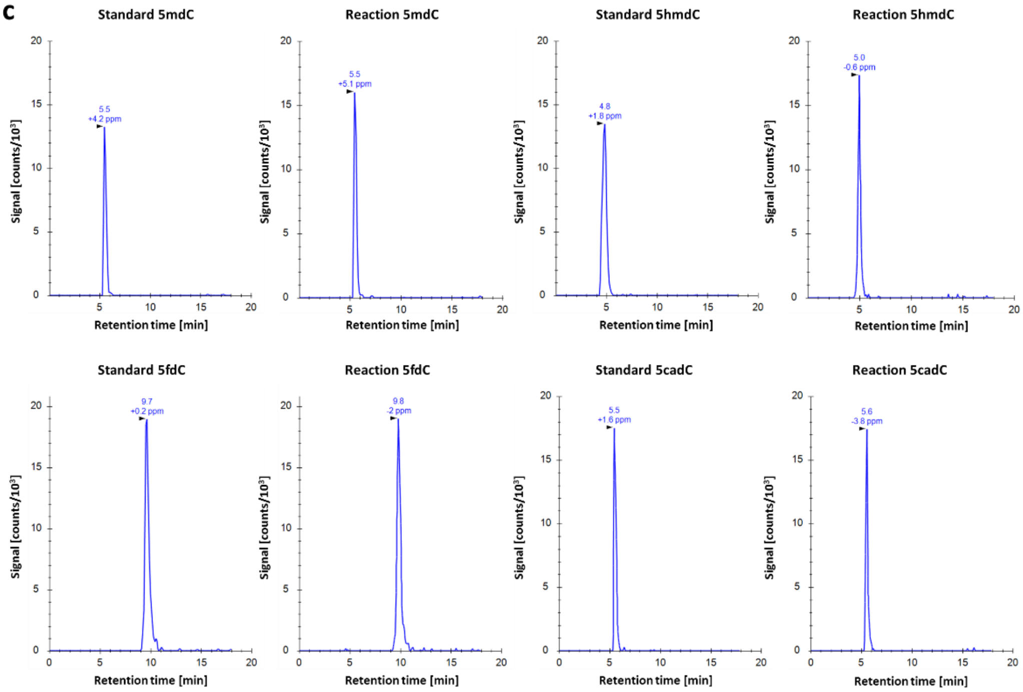

Supplementary Figure 7: Fitting of kinetic data requires a processive reaction step. Two examples of fits of reaction progress curves (here for TET1 reacting with the preferred substrate) are shown, which either include a processive step that directly converts 5mC to 5fC and is described by the k_{12} rate constant or not include this step. Note the much worse fit in the right panel. “Exp” refers to the experimental data, “Fit” to the curves showing the best possible fit of the data to the corresponding model.

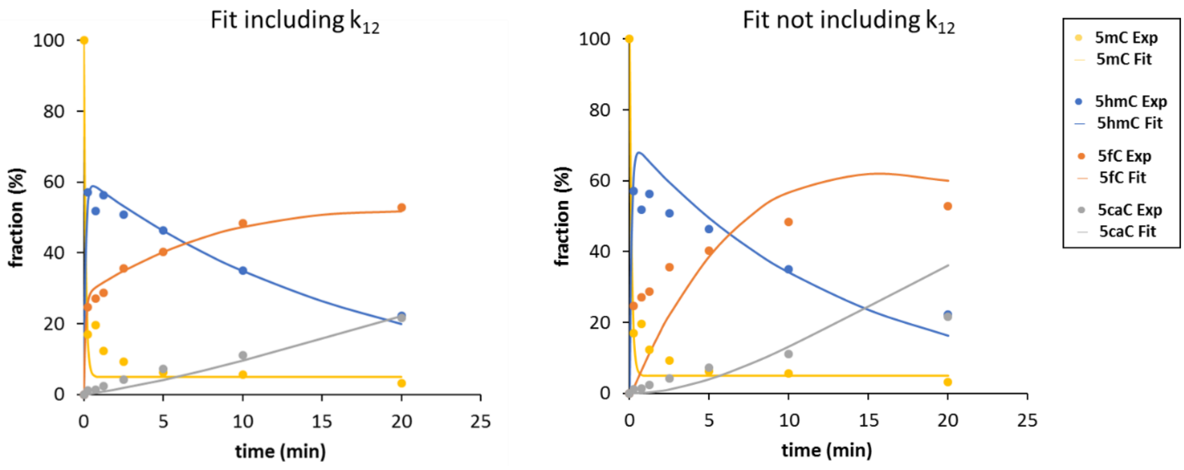

Supplementary Figure 8: Compilation of the correlation of mCpX oxidation flank preferences. Correlation of mCpX -4 to +4 flanking sequence preferences observed in the individual experimental repeats with TET1, TET2 V1 and TET2 V2. The figures show Pearson correlation factors.

TET1		CpG		CpA		CpT		CpC	
		R1	R2	R1	R2	R1	R2	R1	R2
CpG	R1	1.00	0.99	0.83	0.84	0.81	0.84	0.92	0.91
	R2	0.99	1.00	0.84	0.85	0.80	0.84	0.91	0.91
CpA	R1	0.83	0.84	1.00	0.99	0.65	0.68	0.89	0.89
	R2	0.84	0.85	0.99	1.00	0.66	0.69	0.92	0.91
CpT	R1	0.81	0.80	0.65	0.66	1.00	0.90	0.82	0.81
	R2	0.84	0.84	0.68	0.69	0.90	1.00	0.81	0.81
CpC	R1	0.92	0.91	0.89	0.92	0.82	0.81	1.00	0.96
	R2	0.91	0.91	0.89	0.91	0.81	0.81	0.96	1.00

TET2		CpG		CpA		CpT		CpC	
		R1	R2	R1	R2	R1	R2	R1	R2
CpG	R1	1.00	1.00	0.89	0.82	0.62	0.61	0.92	0.92
	R2	1.00	1.00	0.88	0.81	0.62	0.61	0.92	0.92
CpA	R1	0.89	0.88	1.00	0.95	0.45	0.47	0.83	0.81
	R2	0.82	0.81	0.95	1.00	0.45	0.46	0.78	0.76
CpT	R1	0.62	0.62	0.45	0.45	1.00	0.94	0.73	0.71
	R2	0.61	0.61	0.47	0.46	0.94	1.00	0.72	0.70
CpC	R1	0.92	0.92	0.83	0.78	0.73	0.72	1.00	0.99
	R2	0.92	0.92	0.81	0.76	0.71	0.70	0.99	1.00

Supplementary Figure 9: Comparison of genomic NNCGNN 5hmC patterns in lung and liver cells revealing very high similarity. Data were taken from [4]. The Pearson correlation coefficient of both distributions is 0.98. The heatmap scale is between zero and the relative maximum of each sample.

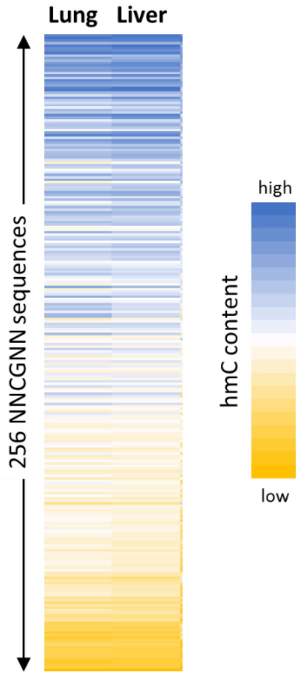

Supplementary Figure 10: Schematic drawing of the TET1 and TET2 enzyme constructs used in this study. For details see the main text.

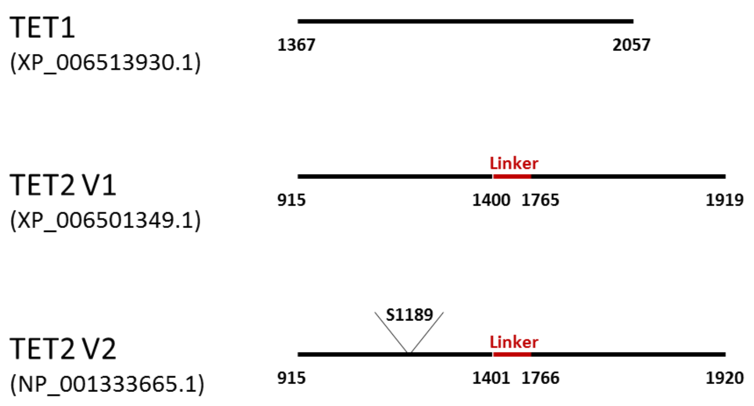

Supplementary Figure 11: Uncropped gels of Figure 3d and e.

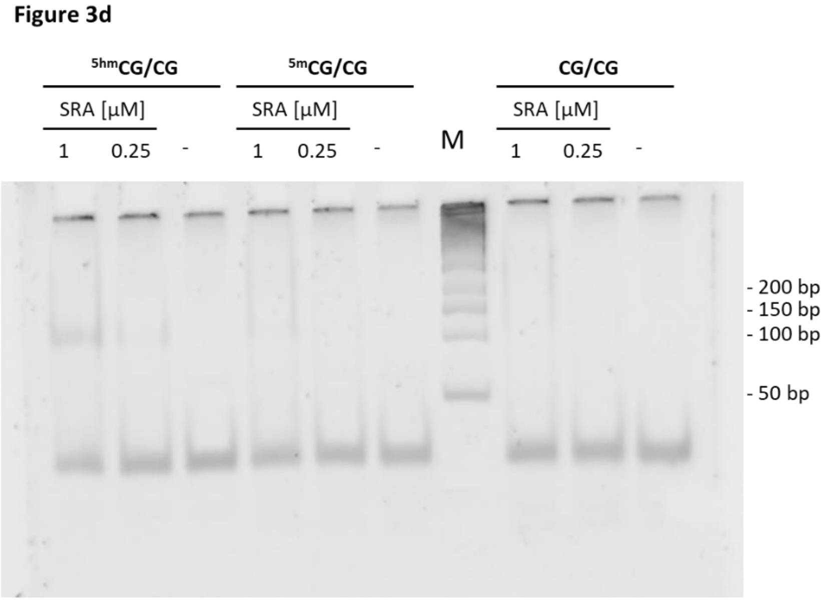

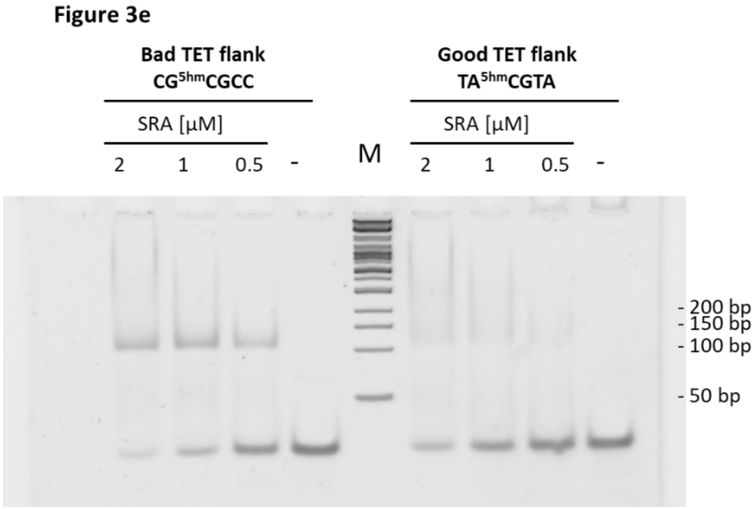

Supplementary References

1 Jeltsch, A., Adam, S., Dukatz, M., Emperle, M. & Bashtrykov, P. Deep enzymology studies on DNA methyltransferases reveal novel connections between flanking sequences and enzyme activity. *J Mol Biol*, 167186 (2021).

2 Adam, S. *et al.* DNA sequence-dependent activity and base flipping mechanisms of DNMT1 regulate genome-wide DNA methylation. *Nature communications* **11**, 3723 (2020).

3 Gao, L. *et al.* Comprehensive structure-function characterization of DNMT3B and DNMT3A reveals distinctive de novo DNA methylation mechanisms. *Nature communications* **11**, 3355 (2020).

4 Li, X., Liu, Y., Salz, T., Hansen, K. D. & Feinberg, A. Whole-genome analysis of the methylome and hydroxymethylome in normal and malignant lung and liver. *Genome Res* **26**, 1730-1741 (2016).
