# Supplementary material for: Flanking sequences influence the activity of TET1 and TET2 methylcytosine dioxygenases and affect genomic 5hmC patterns
Source: Commun Biol. 2022 Jan 24;5:92. doi: 10.1038/s42003-022-03033-4 (PMC8786823; doi:10.1038/s42003-022-03033-4)
Supplement: Supplementary file 3 — Description of Additional Supplementary Files [file 42003_2022_3033_MOESM3_ESM.pdf]

Description of Additional Supplementary Files

File name: Supplementary Data 1
Description: Relative oxidation rates of 5mC and 5hmC substrates in all NNCGNN sites by TET1 and TET2. This table compiles the data shown in Figure 2a.

File name: Supplementary Data 2
Description: Source data and uncropped images.
